# Efficacy of Encorelane in Enhancing Barrier Function and Reducing Aging Signs in Sensitive Skin

**DOI:** 10.1111/jocd.70454

**Published:** 2025-09-15

**Authors:** Lina Wang, Caixia Wang, Liyuan Jiang, Huiping Hu, Yuxuan Wu, Yanan Li, Peiwen Sun

**Affiliations:** ^1^ Research & Innovation Center Proya Cosmetics Co. Ltd Hangzhou China

**Keywords:** anti‐aging, dermo cosmetics, inflammation, sensitive skin, skin barrier function

## Abstract

**Background:**

Sensitive skin requires targeted care to improve barrier function, reduce inflammation, and manage neurovascular reactivity.

**Objective:**

Encorelane, a novel ingredient blend of saccharide isomerate, ribose, and fructooligosaccharides, was evaluated for its efficacy in addressing sensitive skin and visible aging signs.

**Methods:**

In vitro: A 3D epidermal model (EpiKutis) with Sodium Lauryl Sulfate (SLS) induction was used to assess cytokines (interleukin 1α [IL‐1α], interleukin 6 [IL‐6], interleukin 8 [IL‐8], tumor necrosis factor α [TNF‐α], prostaglandin E2 [PGE2]) and barrier markers (filaggrin [FLG], loricrin [LOR], transglutaminase 1 [TGM1]). Hydration was examined via aquaporin 3 (AQP3) expression levels. Anti‐wrinkle efficacy was tested against UVA/UVB exposure, evaluating collagen synthesis and skin matrix components (Collagen types I, III, IV, VII, XVII, Laminin 5, hyaluronic acid [HA], Chondroitin sulfate [CS]). In vivo: A 6‐week double‐blind, half‐face study in 23 sensitive‐skin subjects evaluated the clinical effects of Encorelane versus placebo on repair, redness, firmness, and wrinkles.

**Results:**

In vitro: Compared to the control group, Encorelane significantly reduced the levels of IL‐1α, TNF‐α, IL‐6, IL‐8, and PGE2 (*p* < 0.05), and significantly increased the levels of AQP3, FLG, LOR, TGM1, collagen types I, III, IV, VII, XVII, and laminin 5 (*p* < 0.05). In vivo: Compared to the placebo, Encorelane significantly improved TEWL, R2, F4, and crow's feet wrinkles (*p* < 0.05).

**Conclusion:**

Encorelane effectively targets both inflammation and aging signs, supporting its use as a dermocosmetic solution for sensitive, aging‐prone skin.

## 1 Introduction

Sensitive skin (SS) is commonly characterized by an exaggerated response to physical, chemical, or psychological stimuli, often manifesting with symptoms such as burning, stinging, itching, and tightness, primarily on the face. These subjective symptoms may be accompanied by objective signs like erythema, scaling, and dilated capillaries [1]. The prevalence of sensitive skin is notably high and varies globally due to differences in survey methodologies, with reported rates ranging from 25% to nearly 90% in Europe and around 50% in Australia. In terms of gender distribution, women typically exhibit a higher incidence than men, with reported rates of 22%–51% in the Americas and 40%–56% in Asia [2, 3, 4, 5, 6, 7], with the prevalence among Chinese women at approximately 36% [8, 9]. Moreover, females, especially those post‐30, not only seek to alleviate these symptoms but also aim to improve the cosmetic appearance of their skin, revealing a significant demand for dual‐function cosmetic products.

While several studies have highlighted the efficacy of ingredients such as 4‐t‐butylcyclohexanol, licochalcone A [10, 11], ceramides [12], cholesterol [13], and panthenol [14] in mitigating symptoms and enhancing barrier functions, potent anti‐aging compounds like retinol [15], niacinamide [16], vitamin C [17], and alpha‐hydroxy acids [18] often prove too irritating for sensitive skin types. Despite their benefits, few solutions effectively address both the symptoms of sensitive skin and its aging concerns without triggering further irritation.

In response, we have developed “Encorelane,” a novel ingredient combination comprising saccharide isomerate, ribose, and fructooligosaccharides, designed to improve skin barrier function, minimize inflammation, and reduce vascular reactivity while also delivering significant anti‐aging benefits. This formulation was developed with a deep understanding of the pathogenesis of sensitive skin, which involves a complex interplay of impaired skin barrier function [19], heightened sensory nerve input signals [20], and immune‐inflammatory responses [21] leading to symptoms and long‐term skin changes.

Our approach with Encorelane is based on emerging research that supports the synergistic effects of its primary components. Studies suggest that saccharide isomerate enhances skin hydration and barrier function [22, 23], ribose contributes to cellular energy restoration, improving skin firmness and elasticity [24], and fructooligosaccharides offer anti‐inflammatory benefits [25]. This comprehensive introduction of Encorelane aims to meet the dual needs of managing sensitive skin and combating aging signs, addressing a significant gap in dermatological care.

We have demonstrated the benefits of Encorelane through rigorous in vitro studies and a controlled human clinical trial. The results confirm its effectiveness in enhancing skin barrier integrity, reducing inflammation and redness, and delivering anti‐aging effects. These findings represent a meaningful advancement in the treatment of sensitive skin with aging‐related concerns.

## 2 Materials and Methods

A more detailed description of the experimental protocols, reagent preparations, and antibody information is provided in the Supporting Information.

### 2.1 Test Substances

Prepared at room temperature, Encorelane is composed of Saccharide Isomerate, Ribose, Fructooligosaccharides, 1,2‐Hexanediol, and Trisodium Fructose Diphosphate.

### 2.2 Materials and Grouping

Ex vivo skin tissue from the abdominal region was obtained from surgically excised skin of a healthy 32‐year‐old female volunteer. The collection of ex vivo skin tissue and subsequent treatments were performed by Guangdong BioCell Biotechnology Co. Ltd. The 3D epidermal skin model (EpiKutis) and fibroblasts were also provided by Guangdong BioCell Biotechnology Co. Ltd. The experiments were categorized into the following groups: blank control (BC, without stimulation and no treatment), negative control (NC, with stimulation but no treatment), positive control (PC, with stimulation and treated with experiment‐specific positive standards) and the sample (Encorelane, with stimulation and treated with Encorelane).

### 2.3 Cell Culture and Stimulation

Human primary dermal fibroblasts (HDFs) were seeded into a 6‐well plate and incubated overnight at 37°C with 5% CO_2_ until they reached 40%–60% confluence. Then, 0.3% Encorelane was added to the sample group and incubated for 24 h. HDFs were then exposed to UVA (30 J/cm^2^) for 25 min, followed by another 24 h incubation with or without treatment.

### 2.4 Reconstructed Human Skin Models

EpiKutis models were transferred into a 6‐well plate pre‐filled with EpiGrowth medium and stimulated with 0.2% sodium lauryl sulfate (SLS, Sigma, Cat# 098K0067V, USA). For ELISA assays, the PC group was treated with 0.01% (w/v) dexamethasone (Sigma, Cat# D4902, USA) [26, 27]; for hematoxylin and eosin (H&E) staining and immunofluorescence analysis, the PC group was treated with 50 μM pirinixic acid (Sigma, Cat# 50892–23‐4, USA) [28, 29]. In the ELISA, H&E, and immunofluorescence experiments, the sample group was treated with 6% Encorelane. All treated models were then incubated for 24 h.

### 2.5 Ex‐Vivo Skin Models

The epidermis was placed upward, the dermis downward in a Transwell chamber, which was then transferred to a 6‐well plate with 3.7 mL medium per well and cultured at 37°C in 5% CO_2_ under air‐liquid interface conditions. After 2 days, UVA (30 J/cm^2^) and UVB (50 mJ/cm^2^) irradiation were applied for 4 days. After each session, the medium was replaced, 100 μg/mL Vitamin C (VC, Sigma, Cat# A7506, USA) + 7 μg/mL Vitamin E (VE, Sigma, Cat# 238813, USA) [30, 31] were added to the PC group, and 10% Encorelane was added to the sample group. The culture was then maintained for 3 more days without UV irradiation, with only the treatment applied.

### 2.6 Western Blotting

Based on HDFs, protein expression of phosphorylated‐adenosine monophosphate‐activated protein kinase (P‐AMPK) and adenosine monophosphate‐activated protein kinase (AMPK) was detected using Western blot with P‐AMPK antibody (Abcam, Cat# ab131357, UK) and AMPK antibody (Abcam, Cat# ab32047, UK).

### 2.7 Immunofluorescence Staining

Immunofluorescence was performed on EpiKutis to identify filaggrin (FLG), loricrin (LOR), transglutaminase 1 (TGM1) and aquaporin 3 (AQP3) using FLG antibody (Abcam, Cat# ab218397, UK), LOR antibody (Abcam, Cat# ab198994, UK), TGM1 antibody (Abcam, Cat# ab183351, UK) and AQP3 antibody (Abcam, Cat# ab125129, UK).

Immunofluorescence was performed on ex vivo skin tissue to identify the glycosaminoglycans (GAGs) molecules using biotinylated hyaluronic acid binding protein (HABP, Amsbio, Cat# HKD‐bc41, UK) and chondroitin sulfate (CS) antibody (Abcam, Cat# ab11570, UK).

### 2.8 Immunohistochemical Staining

Immunohistochemistry was performed on ex vivo skin tissue to identify Collagen types I, III, IV, VII, XVII, and Laminin 5 using Collagen I antibody (Proteintech, Cat# 14695–1‐AP, USA), Collagen III antibody (Abcam, Cat# ab23445, UK), Collagen IV antibody (Abcam, Cat# ab6311, UK), Collagen VII antibody (Abcam, Cat# ab309143, UK), Collagen XVII antibody (Abcam, Cat# ab186415, UK) and Laminin 5 antibody (Abcam, Cat# ab14509, UK).

### 2.9 Hematoxylin and Eosin Staining

EpiKutis and ex vivo skin tissue were fixed in 4% paraformaldehyde (Biosharp, Cat# BL539A, China), embedded, sectioned, and stained with H&E.

### 2.10 ELISA Assay

Based on EpiKutis, interleukin 1α (IL‐1α), interleukin 6 (IL‐6), interleukin 8 (IL‐8), tumor necrosis factor α (TNF‐α), and prostaglandin E2 (PGE2) were detected by ELISA (Abcam, Cat# IL‐1α: ab100560, IL‐6: ab46027, IL‐8: ab46032, TNF‐α: ab46087 and PGE2: ab133021, UK) according to manufacturers' instructions.

### 2.11 Human Clinical Study

This randomized, double‐blind, split‐face, controlled trial was conducted in Hangzhou, China, from May to June 2022, involving 23 Chinese women aged 35–59 with sensitive skin.

Participants applied a 30% Encorelane serum to one side of the face and a placebo to the other side, twice daily for 6 weeks. The 30% Encorelane serum and the placebo shared the same formula base, with the exception of the 30% Encorelane component. Evaluations, including image capture, expert assessments, and instrumental measurements, were performed at week 0 (W0), week 2 (W2), week 4 (W4), and week 6 (W6).

Skin hydration was measured using Corneometer CM 825 (Courage + Khazaka, Germany). Transepidermal water loss (TEWL) was measured using Tewameter TM Hex (Courage + Khazaka, Germany), and skin elasticity was measured using Cutometer Dual MPA 580 (Courage + Khazaka, Germany). Facial photographs were taken using the VISIA‐CR system (Canfield, USA).

The study was performed in accordance with the Declaration of Helsinki, and all participants provided written informed consent.

No adverse events were observed or reported by participants during the entire study period, indicating that Encorelane was well tolerated under the test conditions.

### 2.12 Microscopy, Image Analysis and Statistical Testing

Each group was tested in triplicate. Brightfield images were captured using a BX53 microscope (Olympus Industrial; Japan). Fluorescent images were captured using a DM2500 fluorescence microscope (Leica; Japan). Images were analyzed with Image‐Pro Plus software by analyzing a* values and the percentage of the red area.

In vitro data were analyzed using Graph‐Pad Prism, with results presented as Mean ± SEM. Comparisons between groups were analyzed using a *t*‐test, with all statistical analyses being two‐tailed.

$$\text{Inhibition}\;\left(\%\right)=\frac{\text{value of}\;\mathrm{NC}\;\text{group}-\text{value of Encorelane group}}{\text{value of}\;\mathrm{NC}\;\text{group}}\times 100\%$$

$$\text{Improvement}\;\left(\%\right)=\frac{\text{value of}\;\text{Encorelane group}-\text{value of}\;\mathrm{NC}\;\text{group}}{\text{value of}\;\mathrm{NC}\;\text{group}}\times 100\%$$

In vivo data were analyzed by comparing values at each time point to baseline. The Shapiro–Wilk test was applied to assess the normality of data improvement: if *p* > 0.05, data were considered normally distributed, and a paired test was performed; if *p* < 0.05, data were considered non‐normally distributed, and a rank sum test was applied. All statistical tests were one‐tailed, with α set to 0.05.

All confidence intervals reported in this study were calculated at the 95% confidence level.

## 3 Results

### 3.1 In Vitro Results

#### 3.1.1 Encorelane Decreases Inflammatory Markers

**FIGURE 1:**
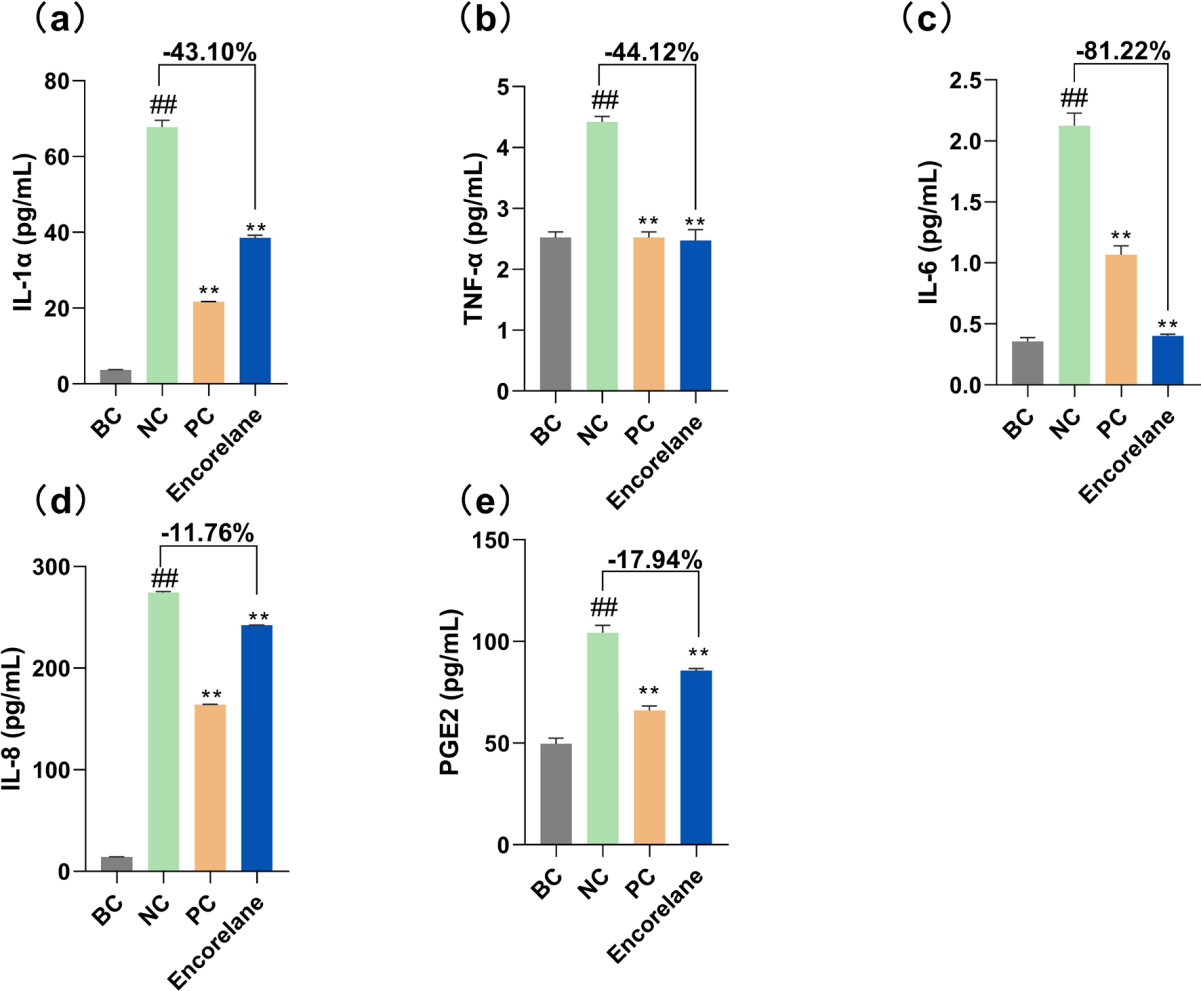
Effects of Encorelane on proinflammatory processes. The application of 6% (v/v) Encorelane to EpiKutis stimulated with 0.2% (m/v) SLS resulted in a decrease in the levels of IL‐1α (a), TNF‐α (b), IL‐6 (c), IL‐8 (d), and PGE2 (e). BC group: Without SLS stimulation, NC group: With 0.2% (m/v) SLS stimulation but without Encorelane treatment, PC group: With both 0.2% (m/v) SLS stimulation and 0.01% (m/v) dexamethasone treatment, and Sample group: With both 0.2% (m/v) SLS stimulation and 6% (v/v) Encorelane treatment. Compared with the BC group, ## means *p* < 0.01. Compared with the NC group, ** means *p* < 0.01. Data are depicted as mean ± SEM.

In the EpiKutis stimulated with 0.2% SLS, Encorelane at 6% (v/v) significantly reduced levels of IL‐1α, TNF‐α, IL‐6, IL‐8, and PGE2 compared to the control group (NC), with inhibition rates of 43.10%, 44.12%, 81.22%, 11.76%, and 17.94% respectively (Figure 1).

#### 3.1.2 Encorelane Enhances Skin Barrier Markers

**FIGURE 2:**
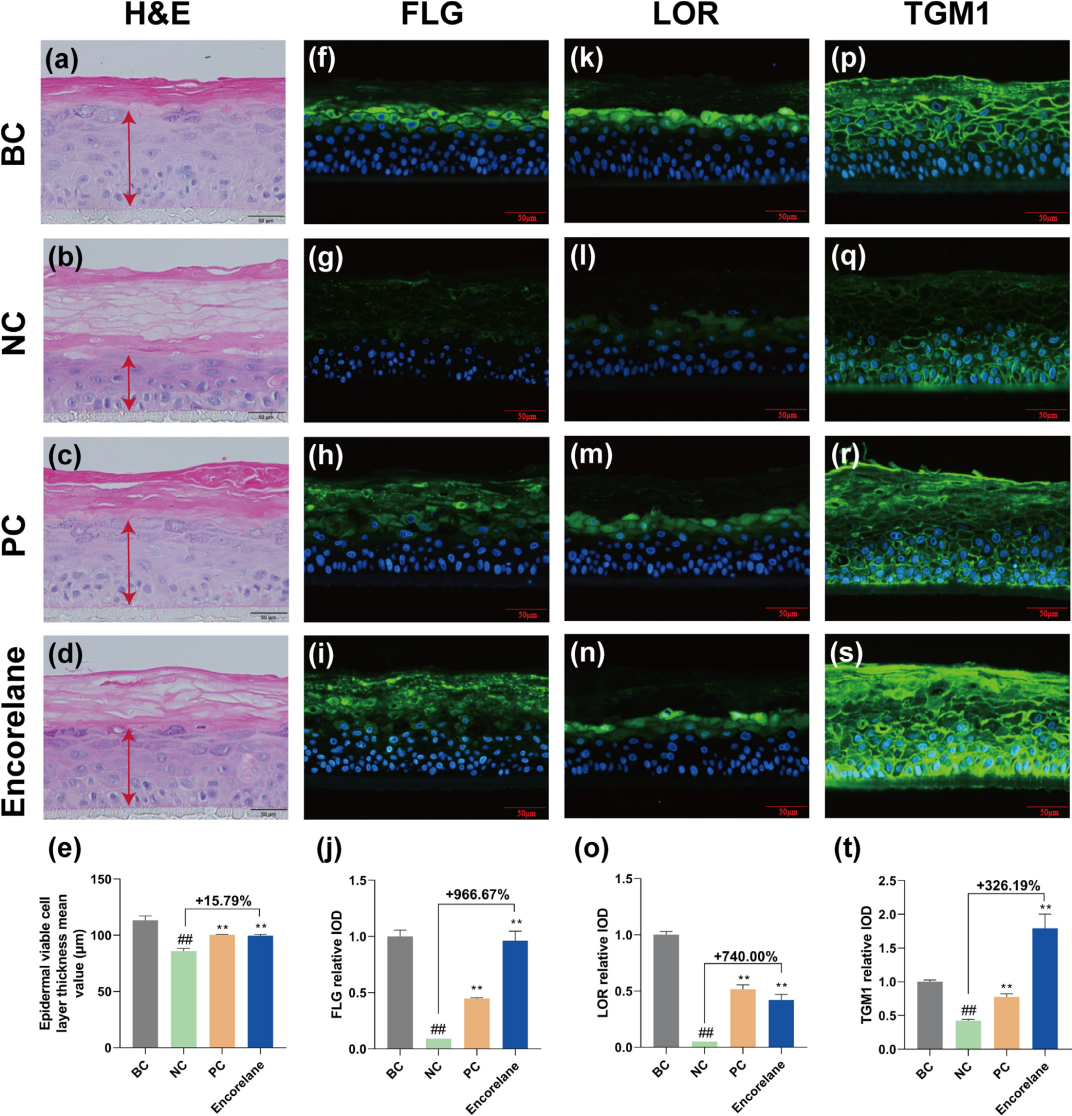
Effects of Encorelane on barrier‐related molecules. The effects of 0.2% (m/v) SLS stimulation on EpiKutis were assessed by H&E staining and immunofluorescence staining to evaluate changes in tissue morphology, FLG, LOR, and TGM1 levels. (a–d) Tissue morphology images were captured using an upright microscope after H&E staining showed a reduction in the number of living cell layers (red arrows) and the formation of vacuoles following SLS stimulation, which were significantly improved after 6% (v/v) Encorelane treatment. (e) Data on the thickness of the viable epidermal cell layers; fluorescence microscopy analysis of FLG, LOR, and TGM1 expression in the skin model, with blue fluorescence representing nuclei and green fluorescence representing FLG (f–i), LOR (k–n), and TGM1 (p–s). (j) FLG, (o) LOR, and (t) TGM1 relative IOD average values. Scale Bar = 50 μm. BC group: Without SLS stimulation, NC group: With 0.2% (m/v) SLS stimulation but without Encorelane treatment, PC group: With both 0.2% (m/v) SLS stimulation and 50 μM pirinixic acid treatment, and Sample group: With both 0.2% (m/v) SLS stimulation and 6% (v/v) Encorelane treatment. Compared with the BC group, ## means *p* < 0.01. Compared with the NC group, ** means *p* < 0.01. Data are depicted as mean ± SEM. IOD: Integral optical density.

Histological analysis using H&E staining revealed a significant increase in viable epidermal cell layer thickness by 15.79% and a reduction in vacuolation in the Encorelane‐treated group compared to NC. Immunofluorescence confirmed significant increases in FLG, LOR, and TGM1 by 966.67%, 740.00%, and 326.19%, respectively (Figure 2).

#### 3.1.3 Encorelane Increases AQP3 Expressions

**FIGURE 3:**
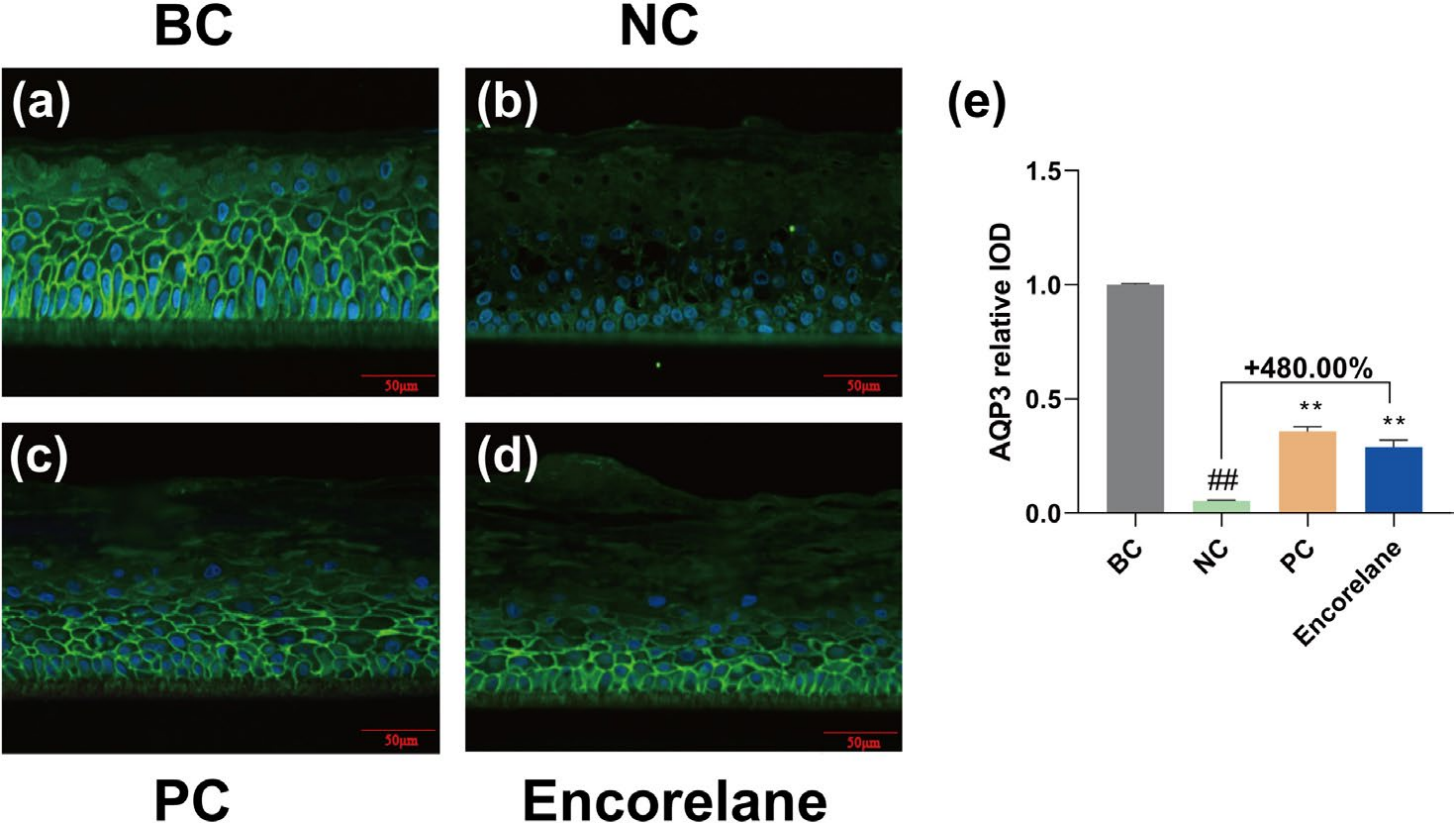
Effects of Encorelane treatment on AQP3 expression. Immunofluorescence staining was performed, and images were captured using a fluorescence microscope to observe AQP3 levels in the EpiKutis under three conditions: BC group: Without SLS stimulation (a), NC group: With 0.2% (m/v) SLS stimulation but without Encorelane treatment (b), PC group: With both 0.2% (m/v) SLS stimulation and 50 μM pirinixic acid treatment (c) and Sample group: With both 0.2% (m/v) SLS stimulation and 6% (v/v) Encorelane treatment (d). Blue fluorescence represents the nuclei, while green fluorescence represents AQP3. Analysis showed that AQP3 levels significantly increased after 6% (v/v) Encorelane treatment (e). Scale Bar = 50 μm. Compared with the BC group, ## means *p* < 0.01. Compared with the NC group, ** means *p* < 0.01. Data are depicted as mean ± SEM. IOD, Integral optical density.

AQP3 levels in the Encorelane‐treated cells showed a 480.00% increase, indicating enhanced hydration capabilities (Figure 3).

#### 3.1.4 Encorelane Increases Collagen Synthesis and Skin Matrix Components

**FIGURE 4:**
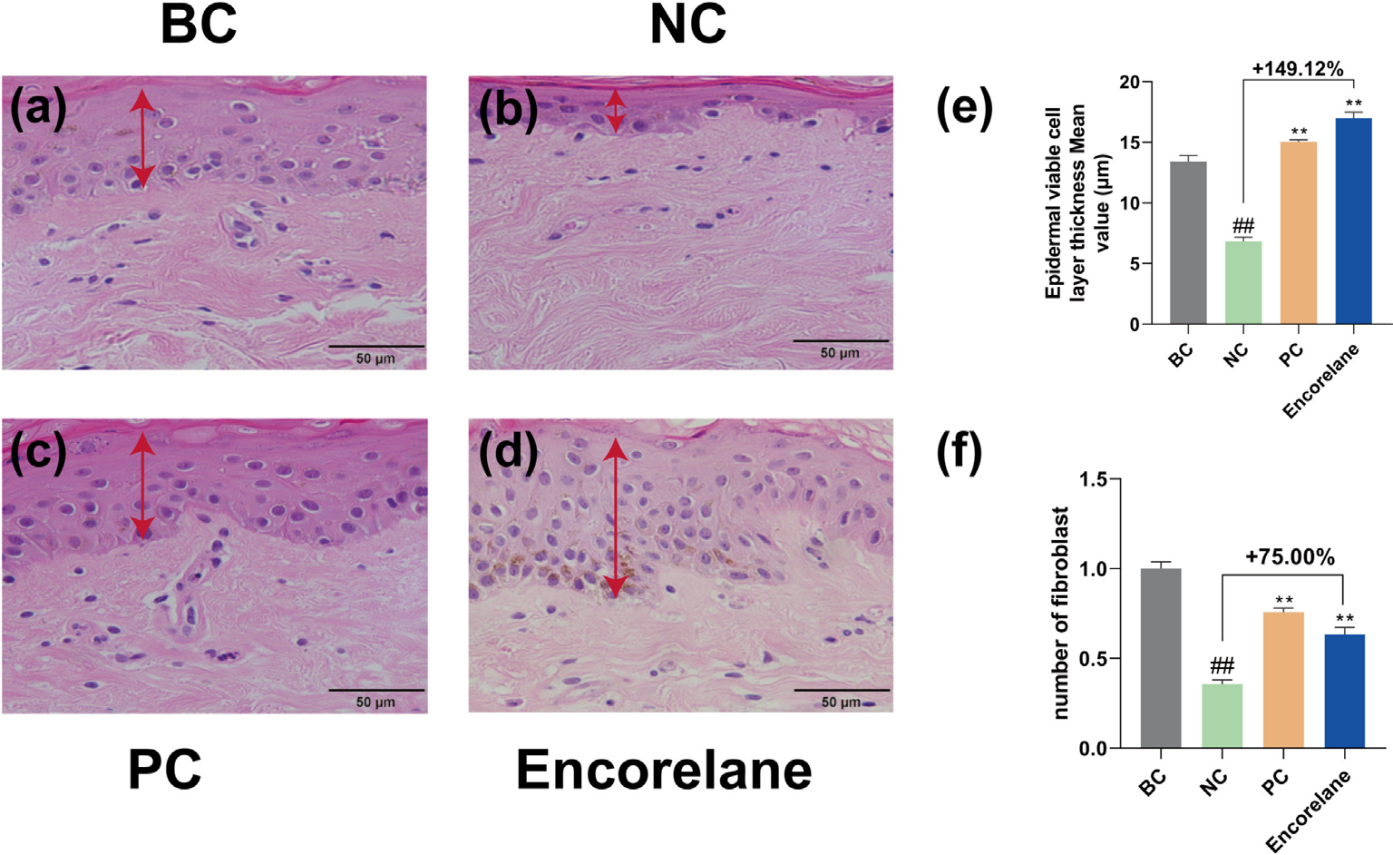
Effects of Encorelane treatment on epidermal tissue morphology. H&E‐stained tissue morphology images were captured using an upright microscope to observe the thickness of the viable epidermal cell layer in ex vivo human skin under four conditions: BC group: Without combined UVA/UVB exposure (a), NC group: With combined UVA/UVB exposure but without Encorelane treatment (b), PC group: With combined UVA/UVB exposure and 100 μg/mL Vitamin C (VC) + 7 μg/mL Vitamin E (VE) treatment (c), and Sample group: With combined UVA/UVB exposure and 10% (v/v) Encorelane treatment (d). The red arrows indicate the thickness of the viable epidermal cell layer. H&E staining analysis showed a significant increase in the thickness of the viable epidermal cell layer (e) and the density of fibroblasts in the dermal layer (f) after 10% (v/v) Encorelane treatment. Scale Bar = 50 μm. Compared with the BC group, ## means *p* < 0.01. Compared with the NC group, ** means *p* < 0.01. Data are depicted as mean ± SEM.

**FIGURE 5:**
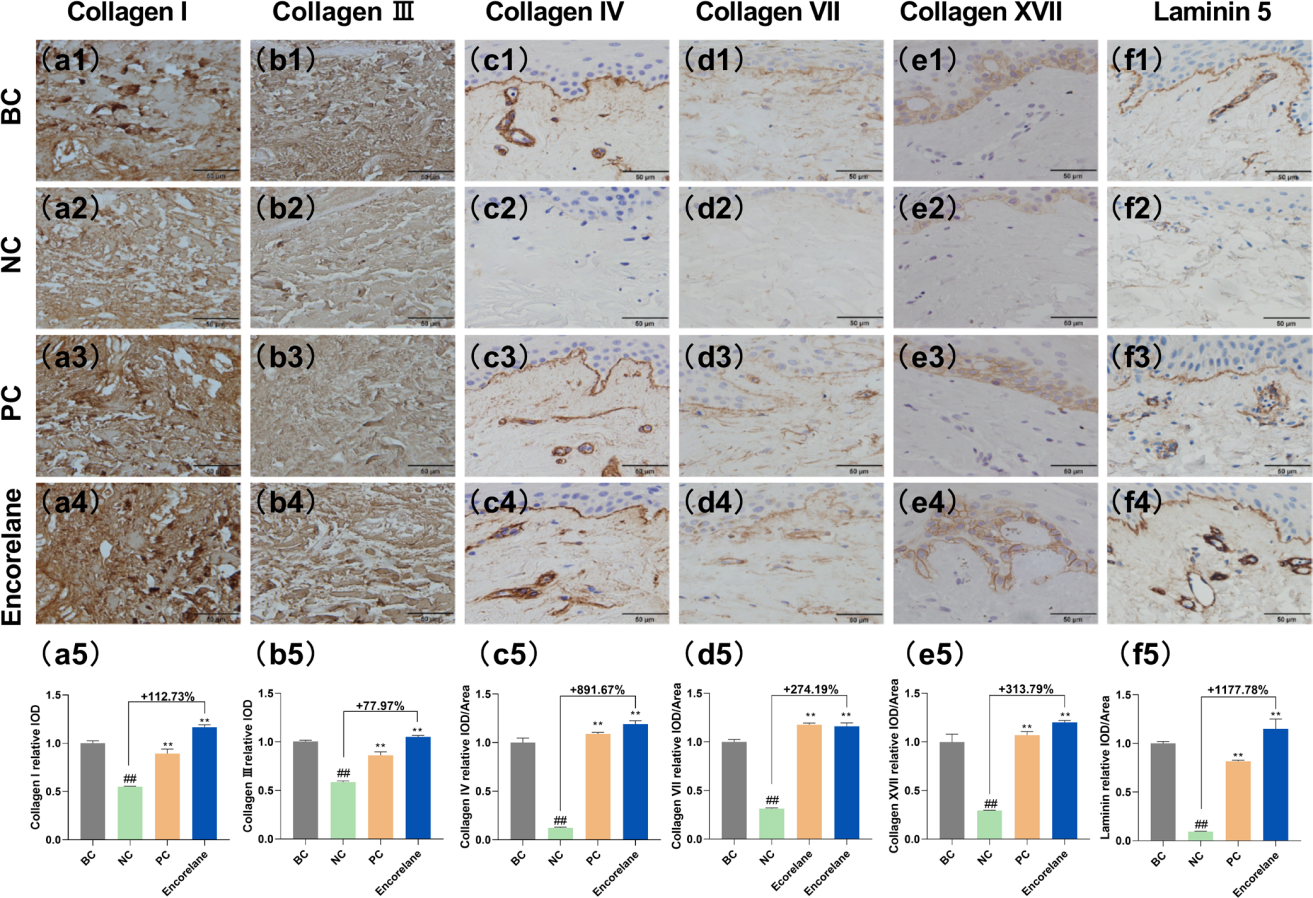
Effects of Encorelane treatment on collagen synthesis. Immunohistochemical staining images were captured using an upright microscope, showed the expression of collagen I (a1–a4), collagen III (b1–b4), collagen IV (c1–c4), collagen VII (d1–d4), collagen XVII (e1–e4), and laminin 5 (f1–f4) in samples under four conditions: BC group: Without combined UVA/UVB exposure (a1, b1, c1, d1, e1, and f1), NC group: With combined UVA/UVB exposure but without Encorelane treatment (a2, b2, c2, d2, e2, and f2), PC group: With both combined UVA/UVB exposure and 100 μg/mL Vitamin C (VC) + 7 μg/mL Vitamin E (VE) treatment (a3, b3, c3, d3, e3, and f3) and Sample group: With both combined UVA/UVB exposure and 10% (v/v) Encorelane treatment (a4, b4, c4, d4, e4, and f4). Brown staining indicates collagen. Analysis showed a significant increase in collagen I (a5), collagen III (b5), collagen IV (c5), collagen VII (d5), collagen XVII (e5), and laminin 5 (f5) after 10% (v/v) Encorelane treatment. Scale Bar = 50 μm. Compared with the BC group, ## means *p* < 0.01. Compared with the NC group, ** means *p* < 0.01. Data are depicted as mean ± SEM. IOD, Integral optical density.

**FIGURE 6:**
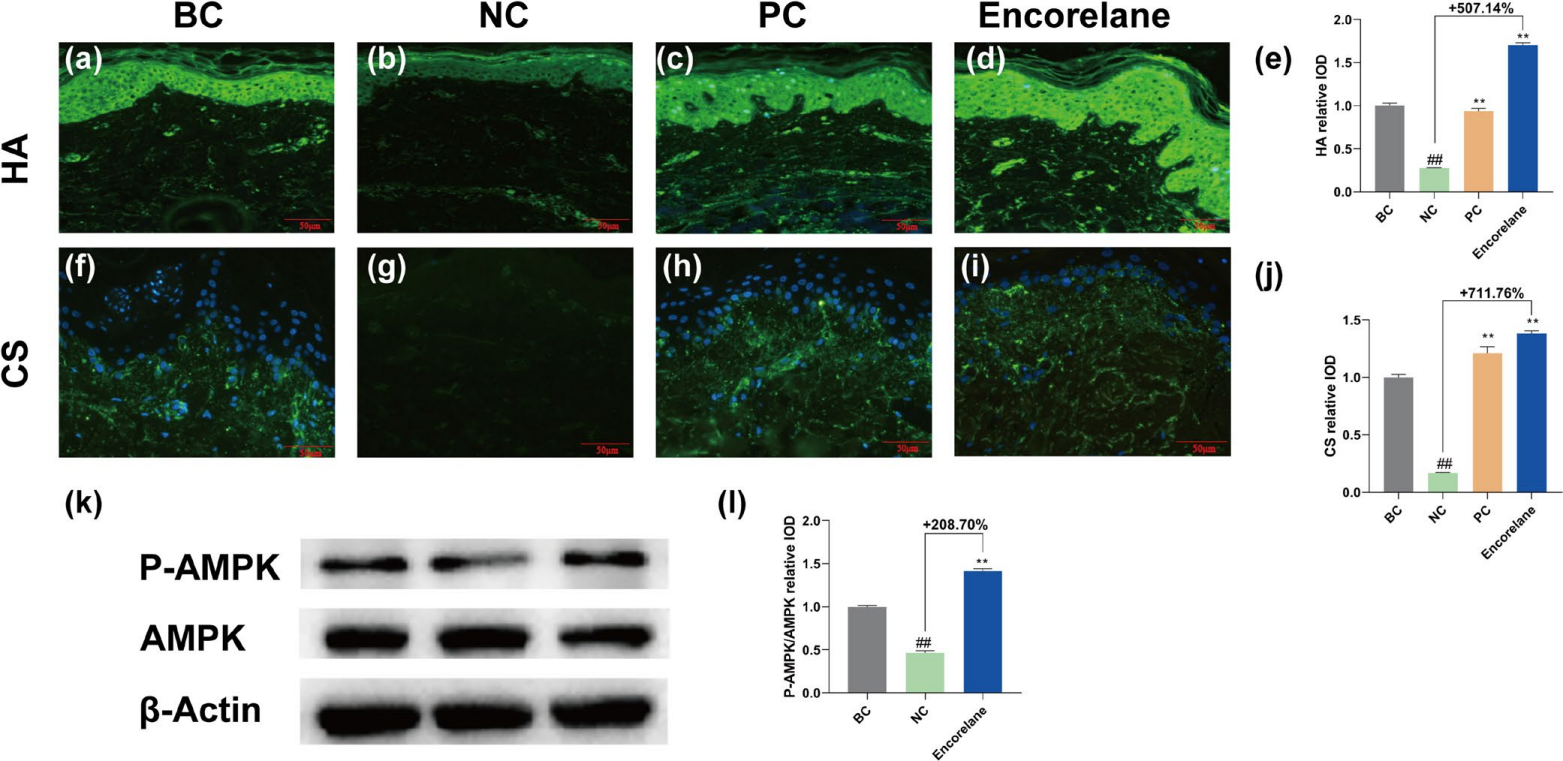
Effects of Encorelane treatment on GAGs Molecules and P‐AMPK/AMPK Ratio. Immunofluorescence staining was performed, and images were captured using a fluorescence microscope to observe the expression of hyaluronic acid (HA) (a–d) and Chondroitin sulfate (CS) (f–i) in samples under four conditions: BC group: Without combined UVA/UVB exposure (a, f), NC group: With combined UVA/UVB exposure but without Encorelane treatment (b, g), PC group: With combined UVA/UVB exposure and 100 μg/mL Vitamin C (VC) + 7 μg/mL Vitamin E (VE) treatment (c, h) and Sample group: With both combined UVA/UVB exposure and 10% (v/v) Encorelane treatment (d, i). Green fluorescence represents HA (a–d) or CS (f–i). Analysis of fluorescence intensity showed a significant increase in HA (e) and CS (j) after 10% (v/v) Encorelane treatment. Scale Bar = 50 μm. Western blot was used to detect the levels of P‐AMPK and AMPK (k). The results showed a significant increase in the relative IOD average value of the P‐AMPK/AMPK ratio after 0.3% (v/v) Encorelane treatment. BC group: Without UVA exposure, NC group: With UVA exposure but without Encorelane treatment, and Sample group: With both UVA exposure and 0.3% (v/v) Encorelane treatment (l). Compared with the BC group, ## means *p* < 0.01. Compared with the NC group, ** means *p* < 0.01. Data are depicted as mean ± SEM. IOD: Integral optical density.

Under combined UVA/UVB exposure, Encorelane at 10% (v/v) significantly enhanced epidermal thickness and dermal fibroblast density, with enhancement rates of 149.12% and 75.00%, respectively (Figure 4). Masson's trichrome, immunohistochemistry, and immunofluorescence results reflected a significant increase in collagen types I, III, IV, VII, XVII, and laminin 5 (Figure 5), with corresponding increases in HA (Figure 6a–e) and CS (Figure 6f–j) by 507.14% and 711.76%.

#### 3.1.5 Enhances P‐AMPK/AMPK Ratio

Western blot analysis demonstrated that Encorelane at 0.3% (v/v) significantly raised the P‐AMPK/AMPK ratio by 208.70% (Figure 6k,l).

### 3.2 In Vivo Results

#### 3.2.1 Encorelane Improves Skin Redness and Provides Soothing Effects

**FIGURE 7:**
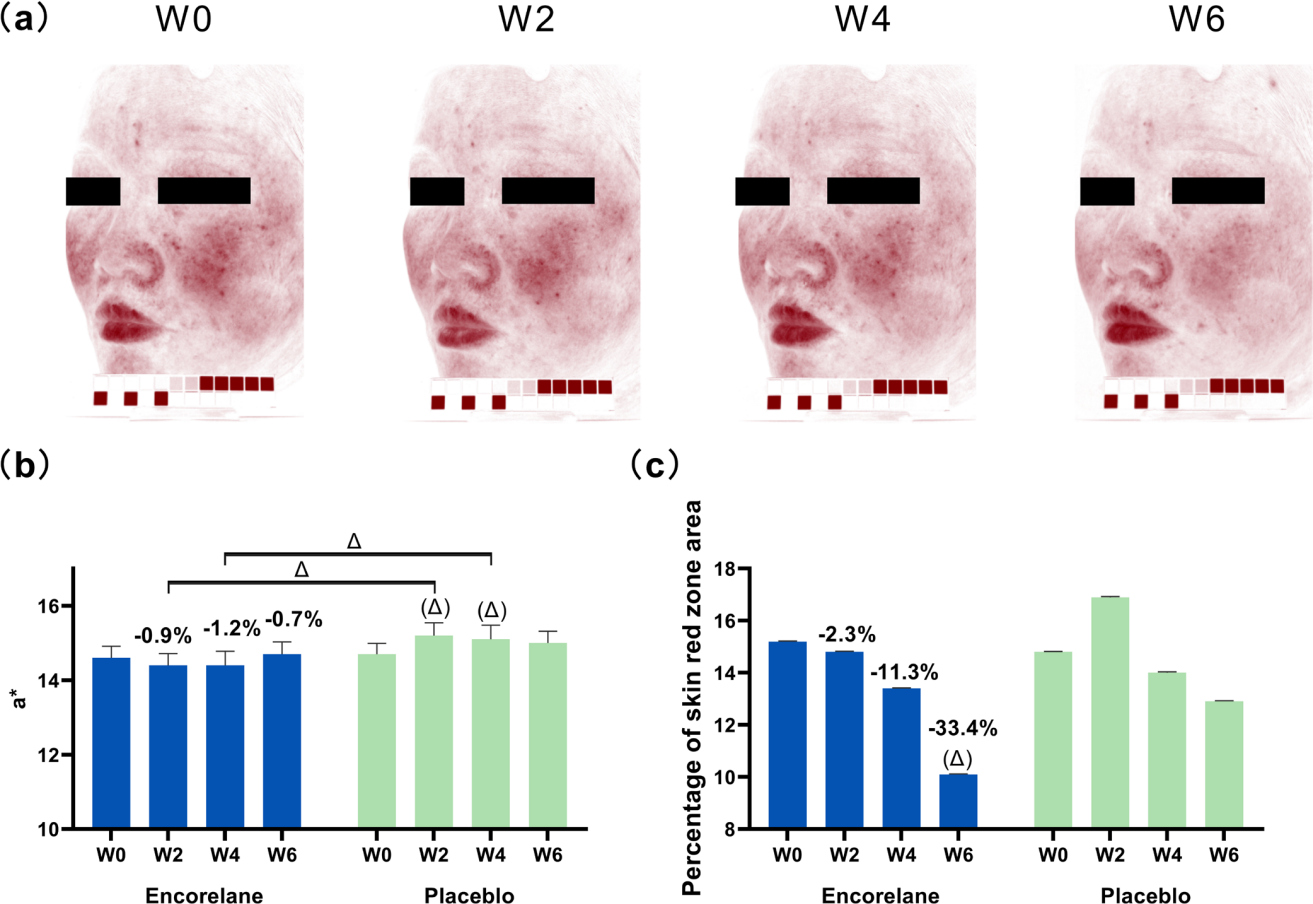
Effects of 30% Encorelane treatment on skin redness (Mean ± SEM). Standard facial redness images were captured using VISIA‐CR to observe improvements in facial skin redness after combined usage of 30% Encorelane serum (a–c). The images are from subject 6, a 35‐year‐old Chinese female (a). After 6 weeks of using 30% Encorelane serum, there was no significant change in a* values, but after 2 and 4 weeks, the a* values showed a directionally significant improvement compared to the placebo (b). After 6 weeks of using 30% Encorelane serum, the percentage of red area was directionally significant reduced compared to baseline (c). Compared to placebo: “Δ”, 0.05 ≤ *p* < 0.1 indicates directionally significant difference. Compared to baseline: “(Δ)”, 0.05 ≤ *p* < 0.1 indicates directionally significant difference.

No significant change in the a* value was noted over 6 weeks; however, the area of redness decreased by 33.4% after 6 weeks of Encorelane use, while the placebo group showed no improvement. Compared to the placebo, the a* value directionally significantly improved after 2 and 4 weeks of using Encorelane, demonstrating its effectiveness in improving skin redness and providing soothing effects (Figure 7).

#### 3.2.2 Encorelane Improves Skin TEWL and Repair Effects

**FIGURE 8:**
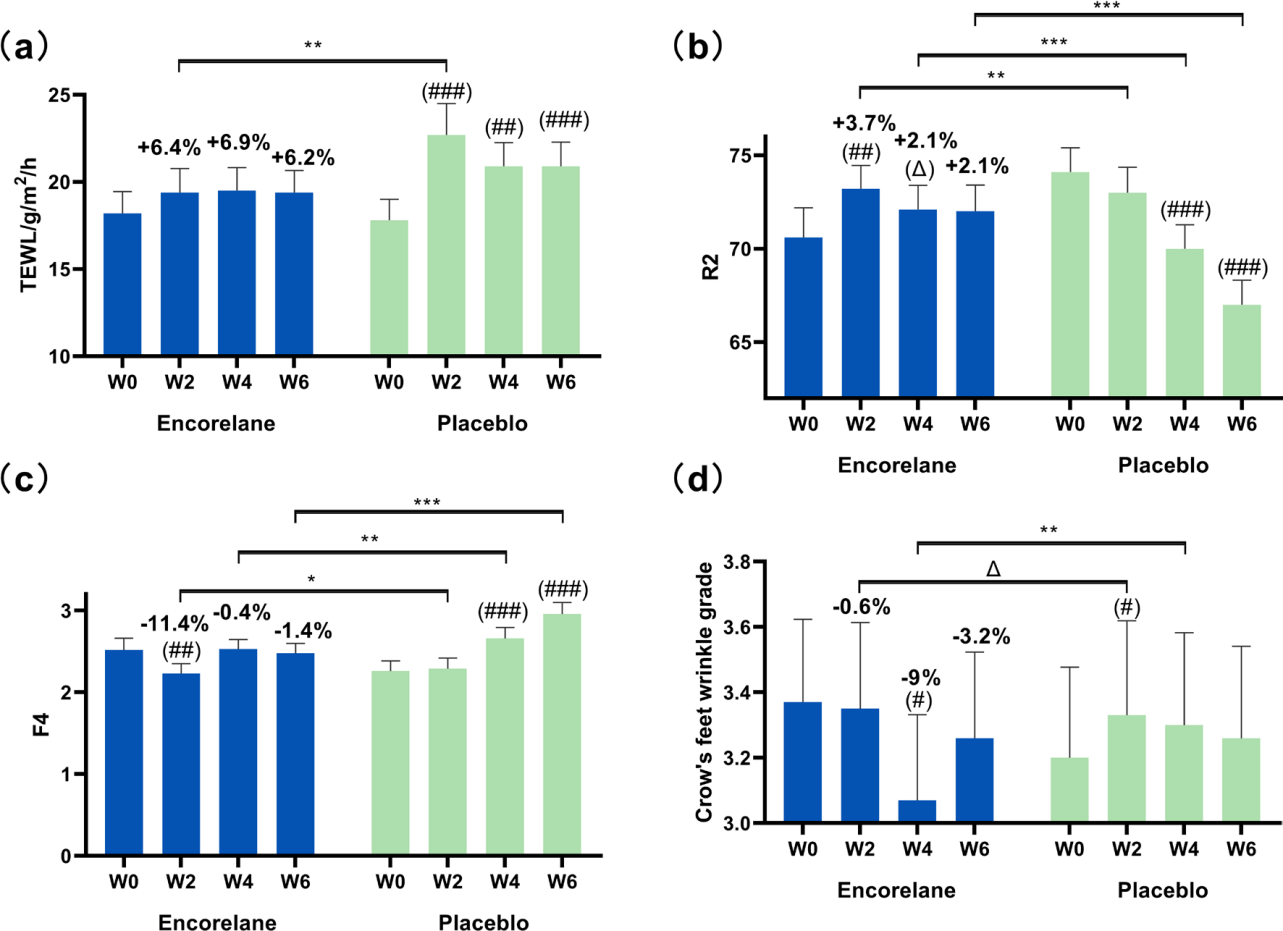
Effects of 30% Encorelane treatment on skin aging (Mean ± SEM). Compared to the placebo, after 2 weeks of using 30% Encorelane serum, there was a highly significant difference in skin transepidermal water loss (TEWL) (a), a highly significant difference in skin elasticity (R2) (b), a significant difference in skin firmness (F4) (c), and a directionally significant difference in crow's feet wrinkle grade (d). After 4 weeks of using 30% Encorelane serum, skin elasticity (R2) showed a very significant difference, skin firmness (F4) exhibited a highly significant difference, and crow's feet wrinkle grade showed a highly significant difference. After 6 weeks of using 30% Encorelane serum, skin elasticity (R2) and firmness (F4) both demonstrated very significant differences. (Compared to placebo: “***”, *p* < 0.001 indicates very significant differences; “**”, 0.001 ≤ *p* < 0.01 indicates highly significant differences; “*”, 0.01 ≤ *p* < 0.05 indicates significant difference; “Δ”, 0.05 ≤ *p* < 0.1 indicates directionally significant difference. Compared to baseline: “(####)”, *p* < 0.001 indicates very significant difference; “(###)”, 0.001 ≤ *p* < 0.01 indicates highly significant difference; “(#)”, 0.01 ≤ *p* < 0.05 indicates significant difference; “(Δ)”, 0.05 ≤ *p* < 0.1 indicates directionally significant difference.)

TEWL measurements showed no significant improvement with Encorelane use at 2, 4, and 6 weeks, but marked worsening in the placebo group by 27.5%, 17.6%, and 17.5%, respectively. Encorelane demonstrated significant improvement in TEWL after 2 weeks compared to placebo (Figure 8a).

#### 3.2.3 Encorelane's Skin Tightening Effects

Encorelane significantly or directionally significantly improved skin elasticity (R2) by +3.7% and +2.1% after 2 and 4 weeks, respectively (Figure 8b), and firmness (F4) by 11.4% after 2 weeks (Figure 8c). In contrast, the placebo group significantly worsened in both metrics over 4 and 6 weeks. Compared to the placebo, both elasticity R2 and firmness F4 showed significant differences after 2, 4, and 6 weeks of using Encorelane, demonstrating its effectiveness in improving skin elasticity and firmness.

#### 3.2.4 Encorelane's Anti‐Wrinkle Effects

Expert assessment showed a 9.0% improvement in crow's feet wrinkles after 4 weeks of Encorelane use. The placebo group's wrinkles worsened by 4.1% after 2 weeks, whereas Encorelane showed directionally significant or significant improvements compared to placebo after 2 and 4 weeks (Figure 8d).

## 4 Discussion

This study examines the hypothesis that Encorelane's distinct formulation enhances skin barrier function and reduces signs of aging. Its efficacy was evaluated through both in vitro and in vivo experiments, focusing on sensitive skin concerns. By integrating dermatological and cosmetic science, this research proposes a practical approach for improving skin health in sensitive, aging populations.

Encorelane's formula contains saccharide isomerate, ribose, and fructooligosaccharides, designed to restore the skin barrier, reduce neurovascular reactivity, manage inflammation, and enhance aesthetic conditions such as wrinkle reduction and skin firming. Based on existing studies, our results corroborate that this combination optimally benefits individuals with sensitive skin by improving barrier functions, reducing TEWL, alleviating inflammation, and improving firmness and wrinkles.

Notably, Dian Andriani Ratna Dewi et al. reported that 5% saccharide isomerate in a moisturizing formulation significantly boosts skin hydration [22], maintaining higher hydration levels post‐treatment compared to standard moisturizers. E. Sze et al. have shown that saccharide isomerates like xylitol possess potent anti‐irritant and anti‐inflammatory properties [23], diminishing TEWL and moderating irritant‐induced increases in dermal blood flow and leukocyte‐endothelial interactions. Additionally, studies by Linda M. Shecterle et al. have demonstrated that supplemental D‐ribose enhances ATP regeneration [24], benefiting fibroblastic activities and clinically improving skin tone and wrinkles. Fujiwara et al. found that oligofructose reduces inflammation and chemotactic factors, alleviating itching [25].

Our systematic in vitro and clinical tests confirmed Encorelane's efficacy and the correlation between its mechanisms and clinical indicators. SLS, an amphiphilic anionic surfactant, disrupts the skin barrier at high concentrations, damaging lipid components and cell membranes, leading to the release of IL‐1α. This cytokine activates nuclear factor kappa‐B (NF‐κB), escalating pro‐inflammatory responses like TNF‐α and PGE2, which manifest as erythema [32, 33]. Our studies utilizing the EpiKutis stimulated with SLS demonstrated Encorelane's potent soothing effects, significantly reducing IL‐1α, TNF‐α, IL‐6, IL‐8, and PGE2, thereby validating its efficacy in alleviating inflammation and enhancing barrier function. Our human clinical test results show that Encorelane significantly improves skin redness and has a soothing effect, consistent with our in vitro results.

Additionally, high concentrations of SLS can damage the skin barrier's “brick‐and‐mortar” structure, primarily constructed from terminal differentiation proteins such as FLG, LOR, and TGM1. Histological analysis via H&E staining shows that SLS exposure loosens the skin barrier and reduces the thickness of the live cell layer, effects that Encorelane in our tests has been shown to mitigate. LOR and FLG are crucial for the integrity and hydration of the skin barrier, forming part of the cornified envelope (CE) and contributing to its stability and moisture retention. TGM1 plays a pivotal role in skin resilience, facilitating the formation of cross‐links that stabilize the skin's structural integrity. Additionally, AQP3, abundantly present in the basal to granular layers of the epidermis, is critical for maintaining skin hydration by facilitating the transport of glycerol and urea. Our experiments utilizing the EpiKutis 3D epidermal skin model stimulated with SLS have effectively demonstrated how Encorelane enhances barrier repair and hydration by positively influencing the expression of FLG, LOR, TGM1, and AQP3, underscoring its potential in improving skin barrier function and overall skin health. Our human clinical test results show that Encorelane significantly improves skin TEWL and has a reparative effect, consistent with our in vitro findings. Skin physiology tests indicate that sensitive skin shows increased TEWL and impaired barrier function [34]. Thus, improving epidermal barrier function is an effective method to improve sensitive skin.

Moreover, UV exposure is a primary factor in skin aging, inducing numerous free radicals that alter extracellular matrix (ECM) components. This leads to the activation of ECM‐degrading enzymes and inhibition of the transforming growth factor β‐Smad (TGFβ‐Smad) signaling pathway, which decreases ECM synthesis. Our microscopic analysis post‐UV exposure reveals notable reductions in epidermal live cell layer thickness, dermal fibroblast density, and collagen content, particularly types IV, VII, and XVII, essential at the dermo‐epidermal junction (DEJ) [35]. These reductions diminish dermo‐epidermal interactions, causing skin laxity. Collagen type I, making up about 80% of the dermis, enhances skin fullness and elasticity, and its increase can mitigate wrinkle formation. Post‐UV, there is an acceleration of keratinocytes differentiation, disrupting their proliferation balance, which thickens the stratum corneum and thins the live cell layer. UV exposure also induces apoptosis in dermal fibroblasts, reducing their density and affecting ECM secretion, leading to symptoms like peripheral sagging [36, 37, 38]. HA, highly concentrated in the skin, aids moisture retention, contributing to ECM stability and resilience against wrinkles. Laminin 5, a significant component of the basement membrane, supports stable epithelial‐cell binding and facilitates cell adhesion and proliferation. CS maintains moisture in connective tissues, enhancing skin plumpness. Encorelane's improvement in the P‐AMPK/AMPK ratio is primarily due to ribose, which influences cellular energy metabolism, impacting cell functions [39]. AMPK, a key energy sensor [40, 41], regulates cellular and systemic energy balance by sensing changes in AMP/ATP and ADP/ATP ratios, promoting energy‐conserving processes. Activation of AMPK, through phosphorylation at its α‐subunit's T172 site (P‐AMPK), reflects its activation level, crucial for ECM secretion. Our in vitro model using fibroblasts and ex vivo skin tissues stimulated with UVA and UVB assesses anti‐wrinkle efficacy by analyzing changes in P‐AMPK, AMPK, tissue morphology, collagen types, and laminin 5. This corresponds with clinical findings showing significant improvements in skin tightness and wrinkle reduction.

In conclusion, this study presents findings from in vitro and human clinical trials indicating that Encorelane may enhance skin barrier function and reduce signs of aging. Encorelane's formulation, a synergistic blend of saccharide isomerate, ribose, and fructooligosaccharides, specifically addresses the complex needs of sensitive skin by reducing neurovascular reactivity and managing inflammation, while also providing anti‐aging benefits. These findings not only highlight the dual functionality of Encorelane in cosmetic dermatology but also demonstrate its potential value in managing of sensitive and aging skin. This research closes a critical gap in dermatological care, offering a scientifically validated solution that enhances skin health and resilience, making a significant contribution to the field and improving patient outcomes in skin aesthetics and health.

One limitation of this study is the relatively small sample size. Although the number of participants did not reach the commonly recommended threshold of 30, valid and analyzable data were obtained from 23 subjects. Additionally, the 6‐week evaluation period may be insufficient to fully capture long‐term effects; thus, extending the observation period will be an important focus of future research. Furthermore, this study did not include a direct comparison between Encorelane and other established active ingredients, which represents another limitation. Comparative studies with such actives will be considered in future investigations to further elucidate the clinical relevance of Encorelane. Because Encorelane targets anti‐aging needs in sensitive skin, we did not include niacinamide or retinoids as split‐face comparators in this first study to avoid confounding irritation in a population prone to reactivity. Future trials in broader populations will consider active‐comparator arms to contextualize efficacy.

## Author Contributions

Lina Wang: Conceptualization, Methodology. Caixia Wang, Liyuan Jiang, Huiping Hu and Yuxuan Wu: Data curation, Writing‐Original draft preparation. Yanan Li and Peiwen Sun: Supervision, Writing‐Review and editing.

## Ethics Statement

Reviewed and approved by Guangdong Biocell IRB; approval #GDLL2022001. The protocol of the in vivo test (PCS‐HCR‐22009) had passed review by the Ethical Commission of Proya Cosmetics Co. Ltd.

## Consent

The patients in this manuscript have given written informed consent to the publication of their case details. Access to biopsy material was in accordance with Chinese law and satisfied the requirements of the local Ethics Committee.

## Conflicts of Interest

The authors declare no conflicts of interest.

## Supporting information


**Data S1:** Materials and Methods.

**Data S2:** Supplementary Tables.

## Data Availability

The datasets used and/or analyzed during the current study are available from the corresponding author on reasonable request.
