# Supplementary material for: Efficacy of Encorelane in Enhancing Barrier Function and Reducing Aging Signs in Sensitive Skin
Source: J Cosmet Dermatol. 2025 Sep 15;24(9):e70454. doi: 10.1111/jocd.70454 (PMC12435122; doi:10.1111/jocd.70454)
Supplement: Supplementary file 1 — Data S1: Materials and Methods. [file JOCD-24-e70454-s002.docx]

**Supplementary Materials and Methods**

**Test substances**

Prepared at room temperature, Encorelane is composed of Saccharide Isomerate, Ribose, Fructooligosaccharides, 1,2-Hexanediol, and Trisodium Fructose Diphosphate.

**Materials and grouping**

Ex vivo skin tissue from the abdominal region was obtained from surgically excised skin of a healthy 32-year-old female volunteer. Once received, the subcutaneous fat was removed, disinfected, trimmed and pre-cultured in turn. The above-mentioned ex vivo skin tissue acquisition and series of treatments were carried out by Guangdong BioCell Biotechnology Co., Ltd. 3D epidermal skin model (EpiKutis), fibroblasts were provided by Guangdong BioCell Biotechnology Co., Ltd.

The experiments were categorized into the following groups: blank control (BC, without stimulation and no treatment), negative control (NC, with stimulation but no treatment), positive control (PC, with stimulation and treated with experiment-specific positive standards) and the sample (Encorelane, with stimulation and treated with Encorelane).

**Cell culture and stimulation**

Human primary dermal fibroblasts (HDFs) were seeded into a 6-well plate and incubated overnight at 37°C with 5% CO_2_. Once the cells reached 40%-60% confluence, 2 mL of culture medium containing 0.3% (v/v) Encorelane was added. The HDFs were then incubated for 24 hours. Following treatment, they were exposed to UVA radiation (30 J/cm²) for 25 minutes, after which incubation continued for another 24 hours with or without treatment.

**Reconstructed human skin models**

EpiKutis models were transferred into 6-well plates containing 0.9 mL of EpiGrowth medium. For the PC group, 12.5 μL of 0.2% (w/v) sodium lauryl sulfate (SLS, Sigma, Cat# 098K0067V, US) solution was applied to the surface. In ELISA assays, the PC group also received 0.01% (w/v) dexamethasone (Sigma, Cat# D4902, US) in the culture medium, while in hematoxylin and eosin (H&E) staining and immunofluorescence analysis, 50 μM pirinixic acid (Sigma, Cat# 50892-23-4, US) was added instead. For the sample group, ELISA, H&E and immunofluorescence experiments received 12.5 μL of 0.4% (w/v) SLS solution and 12.5 μL of 12% (v/v) Encorelane on the surface, achieving final concentrations of 0.2% (w/v) SLS and 6% (v/v) Encorelane. All models were incubated at 37°C with 5% CO_2_ for 24 hours. After incubation, surfaces were rinsed with sterile phosphate-buffered saline (PBS, Solarbio, Cat# P1010, China) and excess liquid was removed.

**Ex-vivo skin models**

The freshly obtained skin tissue was washed with alcohol and PBS, then cut into small circular pieces with a diameter of 0.6 cm. The epidermis was placed upward, the dermis downward in a Transwell chamber, which was then transferred to a 6-well plate with 3.7 mL medium per well, and cultured at 37°C in 5% CO_2_ under air-liquid interface conditions, with daily medium changes. After 2 days, the samples were exposed to UVA (30 J/cm²) and UVB (50 mJ/cm²) irradiation for 4 days After each session, the medium was replaced, 100 µg/mL Vitamin C (VC, Sigma, Cat# A7506, US) + 7 µg/mL Vitamin E (VE, Sigma, Cat# 238813, US) were added to the PC group, and 10% (v/v) Encorelane was added to the sample group. The culture was then maintained for 3 more days without UV irradiation, with only the treatment applied.

**Western blotting**

Methyl thiazolyl tetrazolium (MTT) assays were assessed before experiment to determine working concentration of Encorelane to be tested in vitro with the appropriated cell lines. Cellular proteins were extracted using RIPA lysis buffer (Beyotime, Cat# P0013B, China), and protein concentration was quantified according to the instructions of the BCA Protein Assay Kit (Beyotime, Cat# P0010, China). After protein denaturation, electrophoresis and transfer were performed. The following primary antibodies were used for incubation with the specified dilutions: ACTIN (Proteintech, Cat# 66009-1, US, mouse, dilution 1:1000), Ampk (Abcam, Cat# ab32047, UK, rabbit, dilution 1:1000), p-Ampk (Abcam, Cat# ab131357, UK, rabbit, dilution 1:1000), goat anti-rabbit secondary antibody (Kangwei Century, China, dilution 1:10000), and goat anti-mouse secondary antibody (Kangwei Century, China, dilution 1:10000). Following overnight incubation at 4°C, the membranes were brought to room temperature by incubating for 1 hour. After being washed three times with Tris-buffered saline with Tween-20 (TBST), they were incubated with the secondary antibodies at room temperature for 1 hour. Finally, after three additional washes with TBST, the membranes were exposed.

**Immunofluorescence staining**

The model was fixed with 4% paraformaldehyde (Biosharp, Cat# BL539A, China) for 24 hours, followed by immunofluorescence detection. After deparaffinization and hydration of the sections, antigen retrieval was performed by placing the sections in 0.01 M sodium citrate antigen retrieval solution (diluted 1:50) (Zhonghui Hecai, Cat# PI002, China) under high pressure. Following the quenching of endogenous peroxidase activity, the sections were blocked with serum. They were then incubated with the primary antibody, followed by secondary antibody incubation. After incubation, the sections were washed three times with PBS buffer. Finally, 100 μL of Hoechst 33258 (Amresco, Cat# C1011, US) working solution was added to each section for nuclear staining.

**Immunohistochemical staining**

After treatment, the skin tissue was fixed with 4% paraformaldehyde, embedded, and sectioned for immunohistochemistry. Following deparaffinization and hydration, antigen retrieval was performed. Endogenous peroxidase activity was quenched, and serum blocking was applied. The sections were then incubated with the primary antibody, followed by incubation with the secondary antibody. Subsequently, the ABC complex solution was added and incubated at room temperature. Finally, DAB staining was performed using the horseradish peroxidase DAB staining kit (Sangon Biotech, Cat# C520017-0005, China).

**Hematoxylin and eosin staining**

EpiKutis and the ex vivo skin tissue were fixed with 4% paraformaldehyde, embedded, sectioned, and subjected to H&E staining to observe tissue morphology. After deparaffinization, the sections were stained with hematoxylin for 8 minutes to visualize the nuclei, followed by staining with eosin for 1 minute to stain the cytoplasm, using the Hematoxylin and Eosin Staining Kit (Beyotime, Cat# C0105S, China). Finally, the sections were dehydrated and mounted.

**ELISA assay**

After 24 hours of incubation, the culture medium from the EpiKutis was collected into EP tubes. The samples intended for ELISA analysis were then frozen at -80°C. Detection and analysis of IL-1α, IL-6, IL-8, TNF-α, and PGE2 were performed according to the instructions provided in the IL-1α ELISA kit (Abcam, Cat# ab100560, UK), IL-6 ELISA kit (Abcam, Cat# ab46027, UK), IL-8 ELISA kit (Abcam, Cat# ab46032, UK), TNF-α ELISA kit (Abcam, Cat# ab46087, UK), and PGE2 ELISA kit (Abcam, Cat# ab133021, UK).

**Human clinical study**

A total of 24 healthy female volunteers with sensitive skin were recruited, all of whom provided written informed consent after being informed of the study protocol and potential risks. One participant withdrew due to personal reasons, resulting in 23 subjects who completed the study and were included in the final analysis. The participants were aged between 35 and 59 years, with a mean age of 47 ± 9 years. The participants exhibited facial redness and tested positive in the lactic acid stinging test (score ≥3), reporting itching and stinging sensations on their skin. Expert evaluation confirmed no significant differences between the left and right sides of the participants' faces. Wrinkle grading was conducted on both sides of each participant’s face. Prior to treatment, experts evaluated the crow’s feet wrinkles using the L'Oréal Skin Aging ATLAS (Volume 2, Asian Type). The wrinkle scores between the product-treated side and the placebo-treated side showed no statistically significant differences at baseline.

The trial was conducted as a 6-week, double-blind, split-face study. Encorelane and placebo formulations were applied to either side of the face, randomized using a computer-generated table. Each subject applied product per side twice daily (morning and evening), using standardized pump dispensers. Blinding was maintained for both participants and evaluators throughout the study.

Inclusion criteria: Sensitive skin (positive lactic acid sting test); Able to cooperate well with the study and maintain regular daily routines during the research period; Capable of reading and understanding all contents of the informed consent form, and voluntarily signed the form; Agreed not to use any cosmetics, medications, or health products that could affect the results during the trial; Other applicable inclusion criteria.

Exclusion criteria: Use of antihistamines within the past week or immunosuppressants within the past month; Application of any anti-inflammatory drugs to the test area within the past two months; Presence of unresolved inflammatory skin diseases; Patients with insulin-dependent diabetes; Patients undergoing treatment for asthma or other chronic respiratory diseases; Individuals who received anticancer chemotherapy within the past 6 months; Individuals with immunodeficiency or autoimmune diseases; Pregnant or breastfeeding women; Individuals who have undergone bilateral mastectomy or bilateral axillary lymph node dissection; Participation in other clinical trials; Individuals with highly sensitive constitutions; Non-voluntary participants or those unable to comply with the study requirements; Any other individuals deemed unsuitable for participation by the investigators.

Placebo and Test Formulation Composition:

The placebo formulation used in this study was composed of water, caprylic/capric triglyceride, 1,2-hexanediol, hydroxyacetophenone, and ammonium acryloyldimethyltaurate/VP copolymer, making up 100% of the formulation. The test formulation was identical to the placebo, except that 30% of the water content was replaced with Encorelane, resulting in a final concentration of 30% Encorelane in the test product.
